# The optimal threshold: Baseline serum hepatitis B virus DNA and alanine transaminase levels can predict the 2-Year on-treatment virological response to lamivudine

**Published:** 2011-05-01

**Authors:** Jie Yan, Wen Xie, Qi Wang, Yue Li, Xing Feng, Jun Cheng

**Affiliations:** 1 Center of Liver Diseases, Beijing Ditan Hospital, Capital Medical University, Beijing, China; 2 Institutes of Infectious Diseases, Beijing Ditan Hospital, Capital Medical University, Beijing, China

**Keywords:** Chronic hepatitis B, Lamivudine, Treatment effectiveness, Drug resistance

## Abstract

**Background:**

HBV is still a worldwide health problem. Annually about 0.5-1.2 million patients die of HBV-related diseases such as liver cirrhosis and hepatocellular carcinoma. Lamivudine (LAM) is the first nucleoside analog used in the treatment of chronic hepatitis B. As LAM has been clinically used for a long time, increasing clinical experience has been achieved showing that the resistance mutation rate is relatively high. Numerous studies have also focused on the predictive factors of long-term efficacy of LAM treatment.

**Objectives:**

To determine the optimal cutoff values of baseline hepatitis B virus (HBV) DNA and alanine transaminase (ALT) levels as predictors for the long-term efficacy of LAM treatment in patients with chronic hepatitis B.

**Patients and Methods:**

A total of 163 HBeAg-positive chronic hepatitis B patients receiving LAM treatment were recruited into the present study. Logistic regression analysis was performed to find out the independent predictors of 2-year on-treatment virological response among the baseline parameters. The receiver operating characteristic (ROC) curve was used to evaluate the optimal cutoff values of these independent predictors. The accuracy of the prediction was assessed using the area under curve (AUC) and optimal cutoff values were determined through maximizing the Youden's index.

**Results:**

After 2 years of LAM treatment, undetectable HBV DNA was maintained in 114 (69.9%) patients. LAM-related resistance mutation (YMDD mutation) was detected in 45 (27.6%) patients. Logistic regression analysis indicated that the baseline ALT and HBV DNA levels were the independent predictors of the efficacy. ROC curve analysis suggested the integration parameter derived from the baseline ALT and HBV DNA levels had the maximal predictive value for a 2-year on-treatment virological response. The optimal cutoff values of ALT and HBV DNA were 220 IU/L and 8.2 log10 copies/mL, respectively.

**Conclusions:**

The incidence of LAM-resistant mutations in HBeAg-positive chronic hepatitis B patients may be significantly reduced and long-term efficacy improved when the baseline ALT was greater than 220 IU/L and HBV DNA was less than 8.2 log10 copies/mL.

## Background

Nowadays, hepatitis B virus (HBV) infection is still a worldwide health problem. According to the statistics of World Health Organization (WHO), there are approximately 400 million HBV carriers worldwide, 75% of whom reside in Asia and the Western Pacific. Moreover, annually about 0.5-1.2 million patients die of HBV-related diseases such as liver cirrhosis and hepatocellular carcinoma (HCC) [1]. For patients with chronic hepatitis B (CHB), antiviral therapy is the only treatment which can delay the progression of the disease and improve the long-term prognosis. Therefore, the anti-HBV therapy including interferon-α and nucleos(t)ide analogs has been recommended by the guidelines for the management of CHB [2][3][4][5][6]. Over the last decade, five nucleos(t)ide analogs have been approved by Food and Drug Administration (FDA) for the treatment of CHB, which significantly improved the efficacy. However, the long-term efficacy of nucleos(t)ide analogs is not satisfactory due to drug resistance. Therefore, finding novel predictors for long-term efficacy of anti-viral therapy has been the focus in research on CHB [7][8][9][10][11][12].

The recently revised clinical practice guidelines of European Association for the Study of the Liver (EASL) showed the pre-treatment predictive factors of HBeAg seroconversion were low viral load (HBV DNA below 10(7) IU/mL or 7 log10 IU/mL), high serum ALT levels (above 3-fold ULN) and high activity scores on liver biopsy [5]. However, the cutoff values of HBV DNA and ALT are inconsistent in several studies [13][14][15][16]. Of note, the thresholds of these parameters are frequently determined arbitrarily, according to expert advice or individual experience. The optimal cutoff value achieved from strictly statistical analysis has never been reported. Lamivudine (LAM) is the first nucleoside analog used in the treatment of CHB. In the early years, the mechanisms underlying LAM-resistant mutations and their clinical consequences were not well recognized and LAM was once applied in all CHB patients in China [17][18]. Therefore, the resistance mutation rate increased as high as 20% which affects the long-term efficacy of LAM [19]. As LAM has been clinically used for a long-time, increasing clinical experience has been achieved showing that the resistance mutation rate is relatively high, and thus numerous studies have focused on the predictive factors of long-term efficacy of LAM treatment [7].

## Objectives

The present clinical study was conducted to evaluate the predictive value of baseline HBV DNA and ALT levels in the long-term efficacy of LAM treatment using the receiver operating characteristic (ROC) curve analysis, and to determine the optimal cutoff values of these two parameters.

## Patients and Methods

### Ethics

The present study was an observational study and ethical issues involved privacy during data collection and preservation. The study was approved by the Ethics Committee of Beijing Ditan Hospital, Capital Medical University; all patients gave informed consents.

### Patients and follow-up schedule

A total of 163 CHB patients receiving LAM treatment in Beijing Ditan Hospital, Capital Medical University from July 2003 to November 2006 were recruited into the present study. All patients were HBeAg-positive. All patients met the following diagnostic criteria for HBeAg-positive CHB and had indications for LAM treatment [6]:

patients were positive for serum HBsAg and HBeAg for longer than six months;

HBV DNA ≥ 10(5)copies/mL;

ALT ≥ 2×ULN. All patients were given LAM, 100 mg daily and follow-up was performed once every 12 weeks. The serum HBV DNA level, serological markers of HBV and liver biochemical parameters were monitored.

The primary end-points were as follows: detectable serum HBV DNA by real time polymerase chain reaction (PCR, low limit of quantification, 500 copies/mL) at week 24 or virological breakthrough (defined as detectable HBV DNA in patients who underwent undetectable HBV DNA within the first 24 weeks). The corresponding clinical management was conducted for the patients with detectable serum HBV DNA at week 24. For patients who experienced virological breakthrough, drug resistance mutations were tested in addition to clinical management. If the HBV DNA was maintained undetectable, the follow-up continued for two years.

### Quantitative detection of serum HBV DNA

Real time PCR was used to quantitatively detect serum HBV DNA; to do so PG HBV FQ-PCR kit (PG Biotech, Shenzhen, China) was used. Because the detection range of this system was from 500 to 10(7) copies/mL, the serum was diluted 10(3)-fold for re-examination when the HBV DNA was greater than 10(7) copies/mL.

### Detection of HBV genotypes and reverse transcriptase gene mutations associated with LAM resistance

Direct sequencing was used on products of nested PCR to determine HBV genotypes and resistance mutations within HBV reverse transcriptase gene. The primers used for nested PCR were as follows: P3 (5'-YCTCWSYCAYATCGTCAA-3'), AP3 (5'-GAGMCACAAAGGTTCCAC-3'), Xnp2 (5'-AGGCAGGATAGCCACATT-3'), and X Anp2 (5'-GCACCGAACATGG AGARC-3'). After purification, dideoxy chain-termination method for sequencing DNA was used on products of nested PCR; the primer used was Xnp2. The synthesis of primers and DNA sequencing were performed in Shanghai (Sangon Biological Engineering Technology & Services Co., Ltd, Shanghai, China). DNA sequence analysis was conducted with molecular biological software including DNAstar, ClustalX and GeneDoc.

###  Liver biochemistry and HBV serology

Liver biochemistry including ALT, total bilirubin, albumin, etc was determined every 12 weeks, using an automated chemical analyzer (Beckman Coulter, Fullerton, CA). HBV serological markers, including HBsAg, antibody to HBsAg, HBeAg, and antibody to HBeAg (anti-HBe), were determined every 12 weeks by a micro-particle enzyme immunoassay (Abbot Architect immunoassay system, Abbott Laboratories, Abbott Park, IL) with original reagents.

### Definition of the on-treatment virological response at year 2

On-treatment virological response was considered when the HBV DNA was undetectable by real time PCR after 24-week of LAM treatment which was maintained for two years. Non-response to LAM treatment was considered when 1) HBV DNA was detectable after 24-week LAM therapy; or 2) HBV DNA was undetectable at week 24, but HBV DNA breakthrough was observed thereafter.

### Statistical analysis

The demographics of patients with and without on-treatment virological response were compared by Student's t test for continuous data and x2 test for dichotomous variables. Cumulative rates including HBeAg seroconversion rate and LAM resistance mutation rate were analyzed by Kaplan-Meier procedure. Logistic regression analysis was used to screen independent predictors from the baseline parameters for two-year on-treatment virological response. Receiver operating characteristic (ROC) curve and area under curve (AUC) were used to assess the optimal cutoff values of the predictors through maximizing Youden's index.

A p < 0.05 was considered statistically significant. A 95% confidence interval accompanied all estimates when it was appropriate. All analyses were performed by SPSS version 11.0 (SPSS Inc, Il, USA).

## Results

### Demographic characteristics and baseline parameters

Based on the presence of on-treatment virological response, 163 patients were divided into two groups of "responders" and "non-responders." The demographic characteristics were displayed in Table 1. Significant difference in baseline levels of ALT and HBV DNA was noted between responders and non-responders.

**Table 1:** Demographic characteristics and baseline parameters (Continuous variables are expressed as medians (range)

	**Responders **(No. = 114)	**Non-responders** (No. = 49)	**p-value**
**Sex**			0.997
**Male**	107	46	
**Female**	7	3	
**Age **(years)	31.3 (11.6)	32.0 (10.7)	0.725
**ALT a**(IU/L)	329.7 (298.5)	183.2 (146.4)	< 0.001
**Albumin **(g/L)	40.9 (5.9)	42.7 (5.9)	0.098
**Bilirubin **(μmol/L)	35.6 (50.9)	23.8 (36.9)	0.078
**HBV b DNA ** (log10 copies/mL)	7.4 (0.9)	8.7 (0.8)	< 0.001
**Genotype**			0.74
**B**	32	15	
**C**	82	34	

^a^ ALT: Alanine aminotransferase

^b^ HBV: Hepatitis B virus

### Efficacy and LAM resistance mutation rate after two years of LAM treatment

After two years of LAM treatment, HBV DNA was maintained undetectable in 114 (69.9%) patients which suggested on-treatment virological response. Furthermore, 74 (45.4%) patients had HBeAg loss and 38 (23.3%) had HBeAg seroconversion. HBV DNA breakthrough was observed in 49 (30.1%) patients in the two-year LAM therapy, of whom 45 (27.6%) had LAM-related resistance (YMDD) mutation.

### Optimal logistic regression equation in screening baseline parameters for the prediction of two-year on-treatment virological response

The baseline parameters with a p < 0.1 in the univariate analysis (including ALT, albumin, bilirubin, HBV DNA) served as candidate independent variables; the two-year on-treatment virological response was considered as the outcome (dependent) variable to be used in multivariate logistic regression analysis with forward variable selection technique Table 2. Among the two variables, the relative risk (RR) of baseline ALT was 1.003 (95% CI: 1.001-1.006), suggesting the greater the ALT, the higher the possibility of on-treatment virological response achieved by patients. On the contrary, the RR of baseline HBV DNA was 0.202 (95% CI: 0.117-0.349), suggesting patients with lower-level HBV DNA are more likely to achieve on-treatment virological response.

**Table 2:** The Optimal logistic regression equation of baseline parameters in predicting the two-year on-treatment virological response to LAM treatment

	**β a**	**SE a**	**p a**	**RR a**	**95% CI a**
**ALT a** (IU/L)	0.003	0.001	0.020	1.003	1.001 to 1.006
**HBV a DNA **(log10 copies/mL)	1.601	0.279	< 0.001	0.202	0.117 to 0.349

^a^ β: regression coefficient; SE: standard error; p: significance level; RR: relative risk; 95% CI: 95% confidence interval; ALT: Alanine aminotransferase; HBV: Hepatitis B virus

### Determination of predictive values of baseline levels of ALT and HBV DNA in prediction of two-year on-treatment virological response with ROC curve

**Figure 1:**
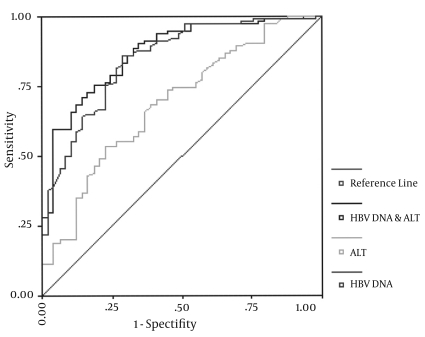
ROC curve of the baseline levels of ALT and HBV DNA in predicting the two-year on-treatment virological response to LAM

The baseline ALT, HBV DNA and integration parameter derived from the optimal logistic regression equation above were used as the predictors for two-year on-treatment virological response to construct an ROC curve (Figure 1). Among these parameters, the integration parameter had the largest AUC of 0.869 followed by HBV DNA (AUC = 0.848). The AUCs of these two parameters were > 0.7 suggesting an acceptable predictive value. The AUC of ALT was only 0.689 indicating a poor predictive value (Table 3).

**Table 3:** AUC of the baseline ALT and HBV DNA in predicting the two-year on-treatment virological response to LAM treatment

	** AUC a**	** SE a**	** P b**	** 95% CI a for AUC a**
**ALT a**	0.689	0.045	<0.001	0.600, 0.777
**HBV a DNA **	0.848	0.033	<0.001	0.783, 0.912
**ALT and HBV DNA c**	0.869	0.030	<0.001	0.811, 0.927

^a^ AUC: Area under the curve; SE: standard error; CI: confidence interval; ALT: Alanine aminotransferase; HBV: Hepatitis B virus

^b^ P: Statistical significance implies that the corresponding AUC was significantly different from 0.5

^c^ Integration parameter derived from the optimal logistic regression equation: 0.003*ALT(IU/L)-1.601*HBV DNA (log10 copies/mL) Integrati on Parameter = 0.003 × ALT(IU/L) − 1.601 × HBV DNA(log10 copies/mL)

### Optimal cutoff values of baseline levels of ALT and HBV DNA in predicting two-year on-treatment virological response

Based on the maximization of Youden's index, the optimal cutoff values of baseline levels of ALT and HBV DNA were 220 IU/L and 8.2 log10 copies/mL, respectively, with the corresponding Youden's index of 0.31 and 0.56, respectively. If the optimal cutoff values were substituted into the optimal logistic regression equation, Youden's index of the integration parameter was 0.39. The corresponding test statistics are listed in Table 4. The possibility of two-year on-treatment virological response was 84.7% when the baseline level of ALT was > 220 IU/L. If the baseline level of HBV DNA was < 8.2 log10 copies/mL, the patients had a probability of 87.3% to achieve a two-year on-treatment virological response. The two-year sustained virological response rate was increased to 95.5% when the ALT was > 220 IU/L and HBV DNA < 8.2 log10 copies/mL.

**Table 4:** Cutoff values of the baseline levels of ALT and HBV DNA in predicting the two-year on-treatment virological response to LAM treatment

	**ALT**	**HBV DNA**	**ALT and HBV DNA**
**Optimal a cutoff value**	220 IU/L	8.2 log10 copies/mL	ALT = 220 IU/L and HBV DNA = 8.2 log10 copies/mL
**Sensitivity**	53.5% (44.4%–62.7%)	84.2% (77.5%–90.9%)	45.6% (36.4%–54.8%)
**Specificity**	77.6% (65.9%–89.2%)	71.4% (58.8%–84.1%)	93.8% (87.2%–100%)
**Likelihood ratio**			
**Positive**	2.38 (1.37–4.12)	2.95 (1.88–4.62)	7.45 (2.44–22.71)
**Negative**	0.60 (0.47–0.77)	0.22 (0.14–0.35)	0.58 (0.48–0.70)
**Predictive value**			
**Positive**	84.7% (76.4%–93.0%)	87.3% (81.0%–93.5%)	95.5% (88.5%–100%)
**Negative**	41.8% (31.6%–51.9%)	66.0% (53.3%–78.8%)	42.6% (33.3%–52.0%)

^a^ The values in parentheses represent the 95% confidence intervals

## Discussion

Increasing studies confirmed that resistance mutation of reverse transcriptase domain of HBV DNA polymerase is the main cause of compromised long-term efficacy of nucleos(t)ide analogs in CHB patients [20][21]. Therefore, predicting the long-term efficacy of nucleos(t)ide analogs is equivalent to predicting presence of a resistance mutation. Among the five nucleos(t)ide analogs used in the treatment of CHB, entecavir has extremely low resistance mutation rate and the five-year resistance mutation rate is only 1.2% when the patients are treated initially with entecavir [22]. Tenofovir disoproxil fumarate is a new drug approved by FDA to be used in the treatment of CHB, and no studies have reported the long term efficacy as well as resistance mutation rate [23]. However, LAM, adefovir dipivoxil and telbivudine have relatively high resistance mutation rates and the two-year resistance mutation rates are 38%, 3% and 17%, respectively [5][24]. Therefore, studies on the predictive factors of long-term efficacy of nucleos(t)ide analogs have paid more attention to these three analogs. The predictive factors in guidelines for the management of CHB patients and expert consensus mainly include the baseline levels of ALT and HBV DNA and decreased level of HBV DNA in the early treatment [5][25][26].

Few studies have been conducted to quantitatively determine the optimal cutoff values of these predictive factors. Yuen, et al. [27] investigated the predictive factors of five-year efficacy of LAM treatment in 74 CHB patients positive for HBeAg. They used the ROC curve to determine the optimal time and HBV DNA level during an early treatment period for the prediction of the response. They drew the conclusions that HBV DNA levels at the 4th week of LAM therapy could predict the five-year ideal response, and that 4 log10 copies/mL was the optimal cutoff value of HBV DNA level at the 4th week of LAM treatment.

We found a maintained HBV DNA response rate of 69.9% and a resistance mutation rate 27.6% after a two-year LAM therapy which was consistent with previous studies. Univariate and multivariate analyses identified the independent predictive factors (baseline ALT and HBV DNA levels) of long-term efficacy of LAM treatment which was also consistent with previously reported. The ROC curve analysis indicated that the integration parameter derived from the baseline levels of ALT and HBV DNA had the maximal predictive value for a two-year on-treatment virological response, with an AUC of 0.869. The cutoff values of baseline levels of ALT and HBV DNA, which were determined by Youden's index maximization, were 220 IU/L and 8.2 log10 copies/mL, respectively. For the CHB patients of our study, there was a probability of 95.5% to achieve on-treatment virological response after two-year LAM treatment when the baseline ALT was > 220 IU/L and HBV DNA was < 8.2 log10 copies/mL. Therefore, favorable long-term efficacy of LAM therapy may be achieved among CHB patients meeting the said criteria. Other drugs, especially those with a low resistance mutation rate, such as entecavir and tenofovir disoproxil fumarate, should be considered for patients who do not fulfill the criteria above [28][29].

These optimal cutoff values were come from a long-term follow-up study in which CHB patients receiving LAM treatment. Whether these cutoff values can be applied to the treatment of CHB patients undergoing other nucleos(t)ide analogs therapy needs to be studied. Furthermore, only few large-scale studies have been conducted to explore the long-term efficacy of LAM treatment in HBeAg-negative CHB patients. These patients were also excluded from the present study. Therefore, the predictive factors of long-term efficacy and the optimal cutoff values in HBeAg-negative CHB patients should be further studied.
